# Reducing Inter-Laboratory Differences between Semen Analyses Using Z Score and Regression Transformations

**DOI:** 10.22074/ijfs.2015.4613

**Published:** 2015-12-23

**Authors:** Esther Leushuis, Alex Wetzels, Jan Willem van der Steeg, Pieternel Steures, Patrick M.M. Bossuyt, Netty van Trooyen, Sjoerd Repping, Frans A.L. van der Horst, Peter G.A. Hompes Hompes, Ben Willem J. Mol, Fulco van der Veen

**Affiliations:** 1 Department of Obstetrics and Gynecology, Vrije Universiteit Medical Center, Amsterdam, The Netherlands; 2 Academic Medical Center, Center for Reproductive Medicine, Amsterdam, The Netherlands; 3 Radboud University Nijmegen Medical Centre, Department of Obstetrics and Gynaecology, Nijmegen, The Netherlands; 4 Jeroen Bosch Ziekenhuis, Department of Obstetrics and Gynaecology, ’s-Hertogenbosch, The Netherlands; 5 University Medical Center Utrecht (UMC Utrecht), Department of Obstetrics and Gynaecology, Utrecht, The Netherlands; 6 Academic Medical Center, Department of Clinical Epidemiology and Biostatistics, Amsterdam, The Netherlands; 7 Department of Clinical Chemical, St. Antonius Hospital, Nieuwegein, The Netherlands; 8 Diagnostic Center SSDZ Reinier De Graaf Group, Department of Clinical Chemistry, Delft, The Netherlands

**Keywords:** Differences, Semen Analysis, Regression, Standardization

## Abstract

**Background:**

Standardization of the semen analysis may improve reproducibility. We assessed variability between laboratories in semen analyses and evaluated whether a transformation using Z scores and regression statistics was able to reduce this variability.

**Materials and Methods:**

We performed a retrospective cohort study. We calculated between-laboratory coefficients of variation (CV_B_) for sperm concentration and for morphology. Subsequently, we standardized the semen analysis results by calculating laboratory specific Z scores, and by using regression. We used analysis of variance for four semen parameters to assess systematic differences between laboratories before and after the transformations, both in the circulation samples and in the samples obtained in the prospective cohort study in the Netherlands between January 2002 and February 2004.

**Results:**

The mean CV_B_was 7% for sperm concentration (range 3 to 13%) and 32% for sperm morphology (range 18 to 51%). The differences between the laboratories were statistically significant for all semen parameters (all P<0.001). Standardization using Z scores did not reduce the differences in semen analysis results between the laboratories (all P<0.001).

**Conclusion:**

There exists large between-laboratory variability for sperm morphology and small, but statistically significant, between-laboratory variation for sperm concentration. Standardization using Z scores does not eliminate between-laboratory variability.

## Introduction

Semen analysis is the cornerstone of the laboratory evaluation of the subfertile male partner (1,2). It is well recognized that the semen analysis demonstrates large between-laboratory variability (3). As a consequence, it is difficult for doctors to interpret and compare the results of semen analyses from different laboratories and this hampers the value of the semen analysis in daily practice. Standardization of the semen analysis might improve reproducibility. A method to standardize across laboratories is Z score transformation, expressing how many standard deviations a semen analysis result is above or below the mean. Z score transformations allow a comparison of observations from different normal distributions. A more conventional method to partition error into systematic and random error is to use regression statistics and then to use the regression coefficients to correct for the systematic error. 

In this study, we assessed systematic differences between laboratories in semen analysis results. We then evaluated whether transformation with Z scores and regression statistics is able to reduce such differences. 

## Materials and Methods

### Circulation samples

In the Netherlands, the National Foundation for Quality Control in Medical Laboratory Diagnostics [Stichting Kwaliteitsbewaking Medische Laboratoriumdiagnostiek (SKML)] sends out samples to the participating laboratories four times a year through the External Quality Assessment Scheme (EQAS). SKML processes and analyses the scores and reports these results to the laboratories. For this study, we collected data of 50 laboratories that had scored the sperm concentration and/or sperm morphology according to local protocols on samples that were regularly distributed by the SKML semen EQAS in 2003. This EQAS was organized according to the International Laboratory Accreditation Cooperation (ILAC-G13) guideline for external quality assessment schemes. All laboratories consented in participating in this study. We circulated 8 samples for sperm concentration and sperm morphology to the participating laboratories. 

## Circulation sample preparation

For concentration measurement, remaining semen of *in vitro* fertilization (IVF) treatments was mixed with Hayem medium (Boom BV, the Netherlands) and stored at 4˚C. Just before a circulation, the different samples were pooled and divided over the batches. The batches were divided over a sufficient number of vials to provide each participating laboratory with a sample of each batch. Dependent of the laboratory, sperm concentration was counted in a Makler, a Burker Turk or an improved Neubauer counting chamber. For sperm morphology, a sufficient number of semen smears was prepared from each sample on microscopic slides. The smears were air dried and distributed over the participants. Ten laboratories scored the sperm morphological parameters according to the 1999 World Health Organization (WHO) criteria and 2 laboratories according to the 1992 WHO criteria (4,5). The procedures with respect to the use of remaining semen were approved by the local Ethical committees. 

## Cohort of subfertile couples

Between January 2002 and February 2004, we included 5,534 couples in a prospective cohort study performed in The Netherlands (6). The local ethics committee of all participating centers gave Institutional Review Board approval. For this analysis, we used data from 2,804 men participating in the prospective cohort in whom the semen analysis was performed by one of the laboratories participating in EQAS (7). In some hospitals, semen analyses were repeated; in our analyses only the results of the first semen analysis were used. 

## Data analysis

To quantify the between laboratories reproducibility in the evaluation of the circulation samples, we calculated between-laboratory coefficients of variation (CV_B_) for sperm concentration and for morphology. The CV_B_ is defined as the ratio of the standard deviation of semen analysis results, returned by the 12 laboratories for a given sample, relative to the mean over all laboratories for that same sample (8) (Addendum 1). CV_B_ values close to zero indicate limited variability between laboratories, hence better reproducibility. With a CV_B_ of 0, all laboratories would return the same result for a single sample. Large CV_B_ indicate low reproducibility.

Addendum 1: concept of the coefficient of variation. 

$$\mathrm{Certificate Of Variation(CV)}=\frac{\sigma }{µ}*100$$

σ : S tan darddeviation

µ : mean

We then calculated Z scores per laboratory, based on the circulation samples (Addendum 2). The Z score represents the difference between a semen analysis result and the laboratory mean, expressed in units of the standard deviation for the results obtained in that laboratory. This Z score can be regarded as a unitless, standardized laboratory result. The Z score is negative when the result is below the mean, positive when above. As the sperm concentration and the sperm morphology were not normally distributed, we applied a natural logarithmic transformation to the sperm concentration values and a square root transformation to the sperm morphology values, before calculating Z scores. To make the Z scores comparable between laboratories, we subsequently adjusted for differences in the laboratory means. 

Addendum 2: concept of the Z score. 

$$Z=\frac{X-µ}{\sigma }$$

$$Z_{l}=\frac{X_{\mathrm{original}}-{µ}_{\mathrm{laboratory}}}{\sigma_{\mathrm{laboratory}}}$$

Z_l_: Z score per laboratory

X_original_ : original semen analysis result

µ_laboratory_: mean per laboratory

σ_laboratory_: standard deviation per laboratory

We used linear regression as an alternative method to adjust for systematic differences between laboratories. We used the mean of the circulation samples as the dependent variable, and the laboratory results as the independent variable. With the laboratory specific regression coefficients, we then adjusted the laboratory results. 

We evaluated whether the Z score transformations and the regression transformations reduced systematic differences in the circulation samples, using analysis of variance. If successful, there should be no significant differences between laboratories after standardization. 

Subsequently, we standardized the semen analysis results from the men in the prospective cohort study using these Z score and regression transformations (Addendum 3). Box plots were constructed to compare semen analysis results per laboratory before and after standardization. Here also, we tested for systematic differences before and after standardization. 

Addendum 3: Standardization of the semen analysis results by Z score transformation. 

$$X_{\mathrm{standardized}}={µ}_{\mathrm{overall}}+\{\frac{X_{\mathrm{original}}-{µ}_{\mathrm{laboratory}}}{\sigma_{\mathrm{laboratory}}}\}*\sigma$$

$$X_{\mathrm{standardized}}={µ}_{\mathrm{overall}}+\{X_{\mathrm{original}}-{µ}_{\mathrm{laboratory}}\}*\{\frac{\sigma_{\mathrm{overall}}}{\sigma_{\mathrm{laboratory}}}\}$$

$$X_{\mathrm{standardized}}=X_{\mathrm{original}}*\{\frac{\sigma_{\mathrm{overall}}}{\sigma_{\mathrm{laboratory}}}\}+\{{µ}_{\mathrm{overall}}-{µ}_{\mathrm{laboratory}}*\{\frac{\sigma_{\mathrm{overall}}}{\sigma_{\mathrm{laboratory}}}\}\}$$

X_standardized_: standardized semen analysis result

µ_overall_: population mean

X_original_: original semen analysis result

µ_laboratory_: mean per laboratory

σ_laboratory_: standard deviation per laboratory

P values under 0.05 were interpreted to indicate statistical significance in all statistical tests. Calculations were performed using The SPSS (SPSS Inc., USA). 

## Results

**Fig.1:**
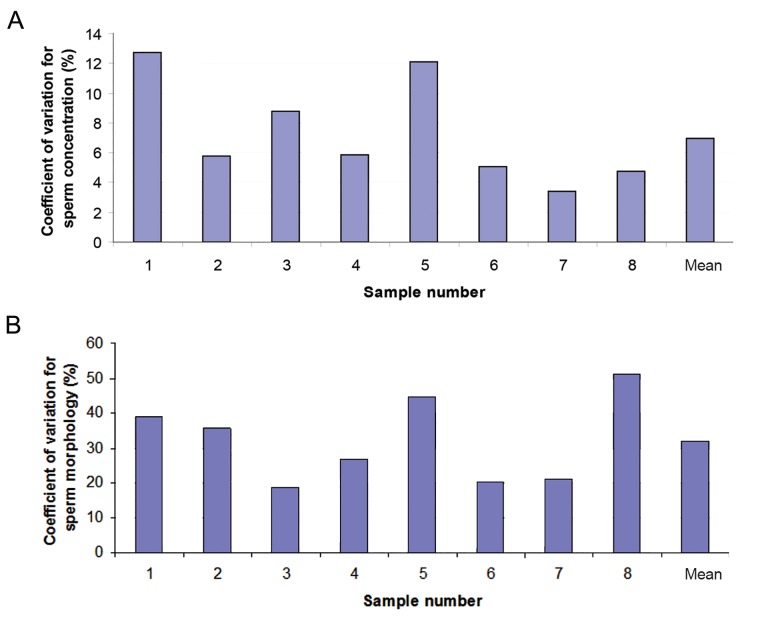
Coefficient of variation for A. Sperm concentration (%) and B. Sperm morphology (%) of 8 samples, evaluated in 12 laboratories.

The mean between-laboratory CV_B_ for the 12 laboratories of the circulation cohort (EQAS) was 7% for concentration (range 3 to 13%) and 32% for morphology (range 18 to 51%). The CV_B_ for sperm concentration and morphology are shown graphically in figure 1A and B, respectively. 

The mean semen analysis results of the men participating in the cohort study before and after standardization with the Z score are shown in table 1. The range for the mean semen volume was 2.6 to 3.9 mL and for the mean sperm motility 30 to 55%. When comparing the ranges for sperm concentration, the range of the mean original semen analyses was 11.6 to 42.1 and 12.1 to 40.4 after regression transformation. For sperm morphology, these ranges were respectively 4 to 31 for the mean original results, 5 to 25 for the mean results after Z score transformation and 9 to 18 for the mean results after regression transformation. 

There were significant systematic differences between laboratories in the results before standardization for the circulation samples, for the four semen parameters: sperm concentration, sperm morphology semen volume and sperm motility (all P<0.001, Table 2). 

We then evaluated whether the Z score transformations reduced systematic differences in the circulation samples. This was not the case (Table 2). 

Transformation with the regression coefficients in the circulation cohort was valid for both semen volume and sperm morphology (Table 2). 

**Fig.2:**
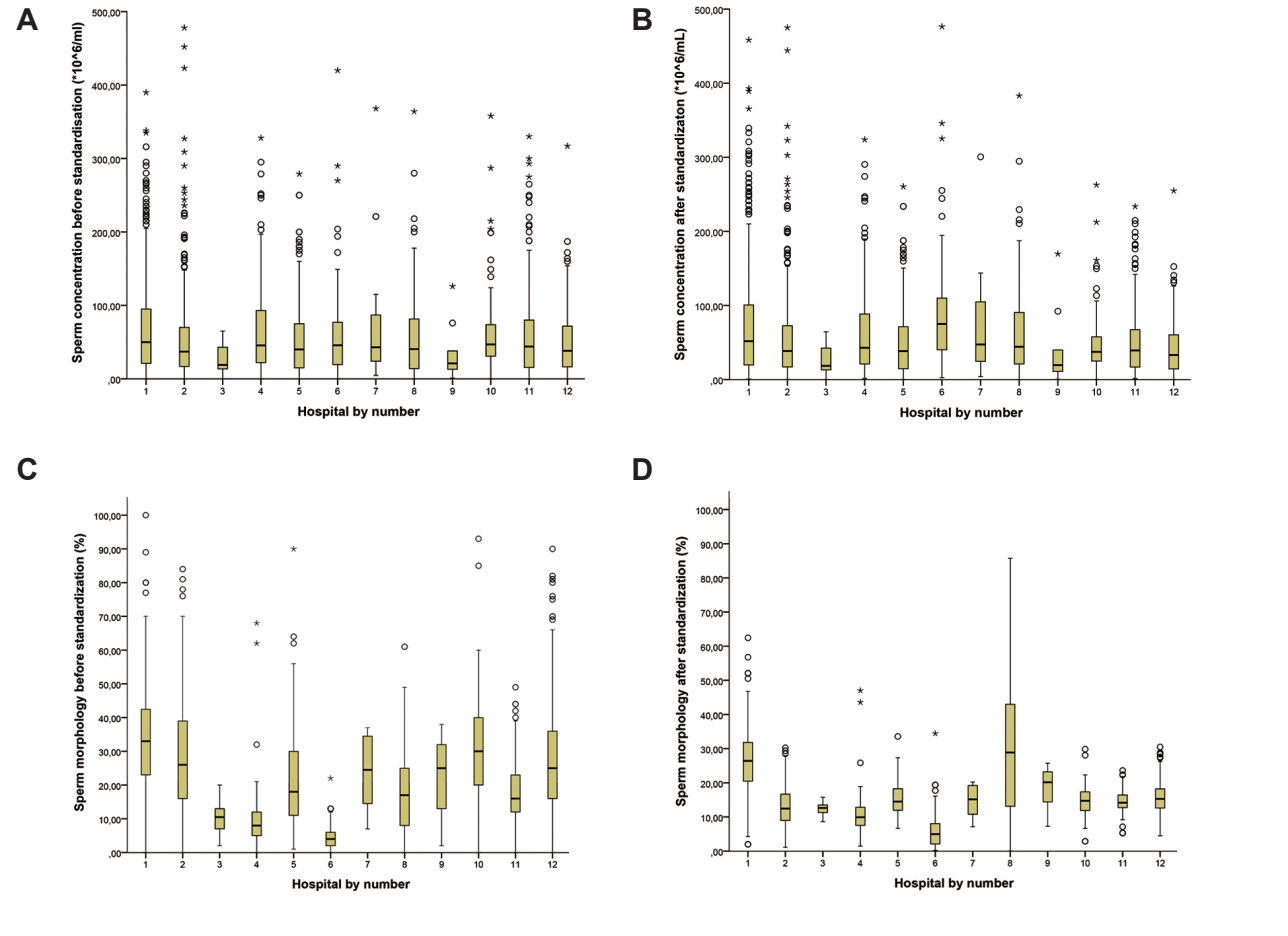
A, B. Box plot of sperm concentration (*10^6^/mL) before and after Z score transformation, C and D. Box plot of sperm morphology (%) before and after Z score transformation. *; Strong outlier, o; Weak outlier, T; Highest value that is no outlier,ₕ ; 25^th^ to 75^th^ percentile, –; Median and ┴; Lowest value that is no outlier.

The, in general, very minor differences for sperm concentration between the values before and after standardization are graphically depicted in figure 2A, B. There was a minimal reduction of the 25^th^ to 75^th^ percentile values, represented by the minimally reduced box sizes. The number of strong and weak outliers did not differ before and after transformation. The box plots for sperm morphology show more differences; the 25^th^ to 75^th^ percentile values are noteworthy smaller after standardization, with fewer outliers, and mean values that are more comparable between laboratories. Visual inspection of the figures showed reduction of the strong and weak outliers (Fig .2C,D). 

When we repeated the analysis of variance after standardization, we still observed significant differences between laboratories for semen volume and sperm morphology for Z score and regression transformations (all P values <0.001, Table 2). 

**Table 1:** Original results of the semen analysis stratified per hospital (n=2,804) that indicates sperm morphology with results after standardization


Hospital	n	Semen volume (ml)	Sperm motility (%)	Sperm concentration (10^6^/ l)	Sperm morphology (%)	
		Original result	Original result	Original result	Original result	Standardized result

1	495		3.1	35	40.9	31	25
2	544		2.7	35	32.5	26	12
3	7		3.8	45	11.6	10	12
4	172		3.5	55	42.1	8	10
5	285		3.5	43	31.8	19	15
6	156		3.3	46	38.5	4	5
7	15		3.7	52	42.1	23	15
8	188		3.8	52	30.9	15	25
9	8		2.6	33	19.9	19	17
10	62		3.9	46	39.3	28	14
11	226		3.7	30	34.4	16	14
12	204		3.0	44	30.9	26	16


**Table 2:** Results of tests for systematic differences in semen analysis results between laboratories in the circulation and the study cohort


	Circulation cohort		Study cohort	
	F	P	F	P

Sperm concentration†				
baseline differences	4.2	<0.001	2.7	0.002
differences after correction by z score	6.8	<0.001	-	-
differences after correction by regression	<0.001	1	3.6	<0.001
Sperm morphology†				
baseline differences	4.1	<0.001	100	<0.001
differences after correction by z score	<0.001	1	137	<0.001
differences after correction by regression	<0.001	1	100	<0.001
Semen volume	12.1	<0.001		
Sperm motility	25	<0.001		


†; Values were transformed to follow a normal distribution, F; F-statistic and P; P values.

## Discussion

In this study, we found systematic differences in results of semen analyses between laboratories for four semen parameters. The between-laboratory coefficient of variation for morphology was large and the between-laboratory variation for concentration was small, but still significant. Standardization of these results by Z score and regression transformations did not reduce these differences between laboratories. 

In this study, we explored the concept of the Z score transformation to standardize differences between laboratories in the semen analysis results and evaluated this in a large multicenter cohort of men from subfertile couples. The Z score transformation procedure for normalizing data is a familiar statistical method in both microarray and psychological studies (9,10), but has never been reported in the field of the semen analysis. 

We used a second more conventional method for the correction of the systematic error, using regression. With the standardization method, we also observed no reduction of the differences between laboratories. 

A first potential limitation of our study was that we had no data to calculate the Z score for sperm motility. A second potential limitation is that outliers in data sets that contain large differences can distort the results. A third potential limitation could be that the random error overshadowed the presence of an effect. Although all measurements are prone to random error, the source of random error in the variability of the semen analysis itself and between laboratories might be difficult to assess. Random errors are unpredictable and scattered about the true value and tend to have null arithmetic mean when a measurement is repeated several times with the same instrument. Another explanation for the inability of both transformation methods to correct for systematic differences might be attributed to differences in populations of the laboratories. Although Z score and regression transformations have the capacity to standardize systematic differences between the laboratories, regardless the source of variability, it is not possible to standardize the effect of the random error and differences in populations. 

With respect to the variability between laboratories, the results of our study are in agreement with a study of 26 semen samples of 26 men that were scored by four teams of specialists from different countries. They reported a reliable comparability for sperm concentration between the four teams, but not for sperm morphology (11). 

## Conclusion

Although all laboratories claimed to follow the WHO recommendations for semen analysis, we established significant differences between laboratories in semen analysis results. The data on intra-laboratory variability was limited in this study. Training and further standardization of all aspects of the semen analysis in combination with internal and external quality control schemes will have to be intensified. This may lead to substantial reductions in intra- and inter-observer variability. In the meantime, laboratories will have to remain repeating semen analyses from patients that were referred with semen analysis results from another laboratory. 
